# Sustained increases in antibiotic prescriptions per primary care consultation for upper respiratory tract infections in England during the COVID-19 pandemic

**DOI:** 10.1093/jacamr/dlad012

**Published:** 2023-02-11

**Authors:** Zheyuan Yang, Sabine Bou-Antoun, Sarah Gerver, Thomas E Cowling, Rachel Freeman

**Affiliations:** Real World Solutions, IQVIA, 37 North Wharf Road, London W2 1AF, UK; Faculty of Epidemiology and Population Health, The London School of Hygiene and Tropical Medicine, Keppel Street, London WC1E 7HT, UK; HCAI, Fungal, AMR, AMU and Sepsis Division, UK Health Security Agency, 61 Colindale Avenue, London NW9 5EQ, UK; HCAI, Fungal, AMR, AMU and Sepsis Division, UK Health Security Agency, 61 Colindale Avenue, London NW9 5EQ, UK; Faculty of Epidemiology and Population Health, The London School of Hygiene and Tropical Medicine, Keppel Street, London WC1E 7HT, UK; Real World Solutions, IQVIA, 37 North Wharf Road, London W2 1AF, UK

## Abstract

**Background:**

The responsible use of existing antimicrobials is essential in reducing the threat posed by antimicrobial resistance (AMR). With the introduction of restrictions during the COVID-19 pandemic, a substantial reduction in face-to-face appointments in general practice was observed. To understand if this shift in healthcare provision has impacted on prescribing practices, we investigated antibiotic prescribing for upper respiratory tract infections (URTI) consultations

**Methods:**

We conducted an interrupted time-series analysis using patient-level primary care data to assess the impact of the COVID-19 pandemic on consultations and antibiotic prescribing for URTI in England.

**Results:**

We estimated an increase of 105.7 antibiotic items per 1000 URTI consultations (95% CI: 65.6–145.8; *P* < 0.001) after national lockdown measures in March 2020, with increases mostly sustained to May 2022.

**Conclusions:**

Overuse of antibiotics is known to be a driver of resistance and it is essential that efforts to reduce inappropriate prescribing continue subsequent to the COVID-19 pandemic. Further work should examine drivers of increased antibiotic prescribing for URTI to inform the development of targeted antibiotic stewardship interventions.

## Introduction

Antimicrobial resistance (AMR) poses a serious threat to global healthcare provision and economic stability.^1^ Prohibitive costs and an unfavourable cost–benefit ratio of developing new antimicrobials has resulted in only two new classes of antibiotics reaching the market since 1962 and a lack of investment in research and development activities.^1,2^ The responsible use of existing antimicrobials is therefore essential in strategies aimed at reducing the threat posed by AMR, and in 2019 the UK government published the AMR National Action Plan (2019–2024),^3^ which included metrics for reducing inappropriate antimicrobial prescribing in primary care.^4^

General practice accounted for 72.7% of all antibiotics prescribed for humans in England in 2020.^5^ A previous study conducted in the UK found that respiratory tract infections (RTIs) were the commonest clinical indication for which antibiotics were inappropriately prescribed;^4^ upper respiratory tract infections (URTIs) are often self-limiting and caused by viruses.

A substantial reduction in face-to-face appointments in general practice was observed immediately following the implementation of measures aimed at reducing the spread of COVID-19 in the UK, with the number of telephone appointments increasing by 270%.^6^

To understand if this shift in healthcare provision has impacted on prescribing practices, we investigated antibiotic prescribing for URTI consultations, comparing antibiotic prescribing for URTI before and during the COVID-19 pandemic in England.

## Methods

IQVIA Medical Research Data (IMRD) contain non-identifiable patient electronic health records (EHRs), collected from general practices.^7,8^ As of 1 June 2022, IMRD included 180 practices and captured data from more than 3.3 million patients. Our study used non-identified EHR data, contributed by practices using EMIS Web clinical software. Data included: year and month of birth (accurate month of birth is only available for patients under 16 years, patients older than 16 years were assigned a proxy month of birth of January); sex; symptoms; diagnosis; drug code; drug name; date of issue; practice location (geographic region); and date of consultation. Remote (telephone and virtual) and face-to-face consultations were included.

We included patients who were diagnosed with an URTI, based on diagnosis and symptom codes, between 1 April 2014 and 31 May 2022. Patients were considered to have received an antibiotic for their URTI if a prescription for an antibiotic was made on the same date as their URTI consultation. Antibiotics were defined using the Anatomical Therapeutic Chemical (ATC) Classification System code J01 (Antibacterials for Systemic Use).^9^ Patients prescribed anti-tuberculosis and anti-leprotic antibiotics on the same date as their URTI consultation were excluded from the analysis.

URTI consultation rates were calculated using numbers of registered patients as the denominator and antibiotic prescribing rates were calculated using numbers of URTI consultations as the denominator. Continuous variables were described by median, IQR, minimum and maximum values. Categorical variables were described by the number and proportion of patients in each category.

Interrupted time-series analysis (ITSA) was used to estimate changes in levels (rates) and trends in antibiotic prescribing over time. Regression models with autoregressive integrated moving average (ARIMA) errors were fitted, using the *auto.arima()* function in the *forecast* package in R (version 4.1.3). The final models were selected using Akaike’s information criterion (AIC) and contained no evidence of residual autocorrelation. Data points were divided into pre-pandemic and pandemic periods, using 23 March 2020 as the cut-point to align with the first national lockdown in England.

Analyses were conducted on the entire patient population of interest and for specific subgroups of age, sex and geographic region.

## Ethics

The use of IMRD for the purpose of medical and public health research and for supplying the data to external researchers for scientifically approved studies under data-sharing agreements has been approved by the NHS Health Research Authority (NHS Research Ethics Committee ref 18/LO/0441) (https://www.iqvia.com/locations/united-kingdom/information-for-members-of-the-public/medical-research-data).

Our study protocol was approved by an independent scientific review committee (SRC) on 30 June 2022 (SRC ref no. 22SRC025). In addition, a combined academic, risk assessment and ethics (CARE) form submitted to the LSHTM MSc Research Ethics Committee was approved on 1 June 2022 (LSHTM MSc Ethics ref. 27304).

## Results

We identified a total of 518 859 patients with at least one URTI consultation in our study period, with 262 851 (50.7%) patients receiving an antibiotic for at least one of these consultations (Table S1, available as Supplementary data at *JAC-AMR* Online). The median age for patients with URTI was 28 years (IQR = 8–48; range = 0–113).

There were 1 079 545 consultations for URTI, and 442 387 (40.9%) antibiotic items prescribed to patients on the same day as their URTI consultation. Consultation rates for URTI showed a steady decrease of 43.5% in the pre-pandemic period from a peak of 24.1 consultations per 1000 registered patients in December 2014 to 13.6 consultations per 1000 registered patients in December 2019. There was a yearly seasonal pattern in the URTI consultation rates, with the peaks in winter and troughs in summer. Consultation rates dropped from 9.6 consultations per 1000 registered patients in February 2020 to 2.3 consultations per 1000 registered patients in April 2020, and remained at similar levels, with the absence of usual seasonal inclines, until March 2021, at which point consultation rates increased (Figure S1).

**Figure 1.**
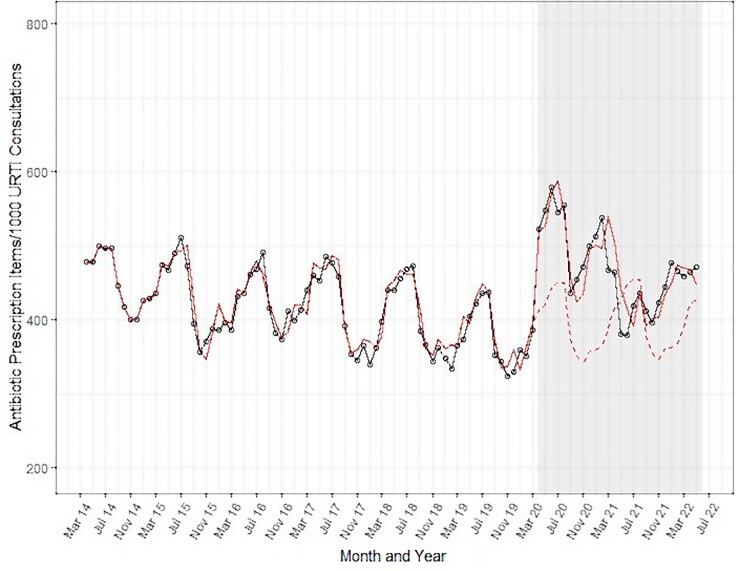
Interrupted time-series analysis of antibiotic prescribing rate for URTI consultations in England, per 1000 URTI consultations, April 2014 to May 2022. (Grey area = COVID-19 pandemic period; black line = observed data; solid red line = predicted values from ARIMA model; dashed red line = counterfactual predicted by ARIMA model in absence of COVID-19 pandemic).

Results from the ITSA revealed that antibiotic prescribing rates for URTI consultations were decreasing in the pre-pandemic period (Figure 1), with peaks in the summer periods coinciding with the troughs in URTI consultation rates. In the pandemic period there was an immediate, significant increase of 105.7 items per 1000 URTI consultations (95% CI: 65.6–145.8; *P* < 0.001)—a 27% increase compared with the predicted value in April 2020, had the pre-pandemic trend continued (Table 1).

**Table 1.** Interrupted time-series analysis results for changes in level and slope of antibiotic prescribing rates for URTI consultations in England, April 2014–May 2022

ARIMA models for antibiotic prescribing rate (prescription items per 1000 URTI consultations)		Estimate of change in level (*P* value)	95% CI	Estimate of change in slope (*P* value)	95% CI
Overall		105.7 (<0.001)	65.56–145.8	−3.1 (0.06)	−6.3 to 0.2
Stratified analyses					
* *Sex	Male	119.3 (<0.001)	75.8–162.7	−2.0 (0.2)	−5.0 to 1.0
	Female	89.78 (<0.001)	46.6–133.0	−1.7 (0.2)	−4.2 to 0.9
* *Age group (years)	0–5	92.3 (<0.001)	56.5–128.2	−3.0 (0.01)	−5.2 to −0.9
	6–11	221.3 (<0.001)	173.1–269.5	−7.8 (<0.001)	−10.7 to −4.8
	12–17	271.7 (<0.001)	223.6–319.8	−4.5 (0.002)	−7.4 to −1.6
	18–59	96.0 (<0.001)	62.6–129.3	1.8 (0.1)	−0.3 to 3.9
	60–74	90.6 (<0.001)	53.6–127.7	1.9 (0.2)	−1.0 to 4.9
	75+	132.6 (<0.001)	84.9–180.3	0.8 (0.7)	−3.9 to 5.5
* *Region	London	173.1 (<0.001)	117.3–228.9	−3.3 (0.1)	−7.6 to 1.0
	Midlands and East of England	159.6 (<0.001)	115.4–203.8	−5.3 (<0.001)	−8.3 to −2.3
	North of England	127.3 (<0.001)	88.0–166.5	−1.4 (0.3)	−4.3 to 1.4
	South of England	67.1 (<0.001)	21.2–113.0	1.5 (0.3)	−1.3 to 4.3

A significant increase in antibiotic prescribing in the pandemic period was seen across all subgroups investigated (Table 1 and Figures S2–S4). The effect of the pandemic on rate changes differed between the age groups, with the greatest increase in prescribing seen in patients aged 12–17 years (Table 1 and Figure S3). Statistically significant decreases in the time trend (slope) were estimated in the pandemic period for age groups 0–5, 6–11 and 12–17 years (Table 1 and Figure S3). In terms of geographic region, increased rate of prescribing by URTI consultation was greatest in London (Table 1 and Figure S4). A statistically significant change in slope was detected for the Midlands and East of England, with the pandemic slope showing a greater decline than the pre-pandemic slope (Table 1 and Figure S4).

## Discussion

Following the introduction of national measures to reduce the spread of COVID-19 in England, consultation rates for URTI reduced substantially. The decrease observed in our study is in line with findings reported in the literature.^6,10,11^ This reduction is likely due to the closure of practices, a shift towards remote appointments,^6^ and patients avoiding accessing healthcare due to the perceived risk of COVID-19 infection. URTI consultation rates did increase as the pandemic progressed, which may be associated with the availability of the COVID-19 vaccine.

It must be noted that total quantity of antibiotics prescribed for respiratory infections in primary care has reduced subsequent to the COVID-19 pandemic,^12^ and the initial sudden increase in rate of prescribing (by URTI consultation) observed may be due to the prioritization of patients with more severe disease. However, the persistence in increased antibiotic prescribing rates for URTI consultations up to May 2022 suggests that there may be a sustained change in antibiotic prescribing. With an estimated 4.95 million deaths associated with antibiotic-resistant bacteria in 2019,^13^ and overuse of antibiotics known to be a driver of resistance,^14–16^ it is imperative that AMR and antimicrobial stewardship remain a global priority.

Diagnostic uncertainties arising as a consequence of conducting clinical assessments remotely may have resulted in prescribers being more likely to prescribe antibiotics for URTI. This was suspected to be the case in the early phase of the pandemic, where the number of antibiotic prescriptions was 6.7% higher than expected given the substantial decrease in consultations.^6^ This is also consistent with the finding that antibiotic prescribing rate is higher in remote consultations than in face-to-face settings.^17^

We acknowledge that our study has several limitations, including that we have only investigated URTI. We focused on URTI due to previous studies identifying higher levels of inappropriate prescribing for this group of infections compared with others.^4^ Additional studies are required to understand the impact of COVID-19 on prescribing for other infections. We were also unable to conduct stratified analyses by consultation setting and believe that this needs to be further explored. Though we have presented analyses by several subgroups we recognize that further work is required to investigate the impact of comorbidities and deprivation on antibiotic prescribing during the COVID-19 pandemic. In addition, the high rate of prescribing in those aged 12–17 years requires further investigation.

The observed persistent increase in antibiotic prescribing for URTI in England post-COVID-19 is concerning and we recommend that other countries consider conducting similar analyses to assess the impact of the pandemic on antibiotic prescribing. Future studies should consider investigating consultation setting on prescribing rates, and patient and prescriber attitudes.

## Supplementary Material

dlad012_Supplementary_Data

## Supplementary data

Figures S1 to S4 and Table S1 are available as Supplementary data at *JAC-AMR* Online.
