# Supplementary material for: Sustained increases in antibiotic prescriptions per primary care consultation for upper respiratory tract infections in England during the COVID-19 pandemic
Source: JAC Antimicrob Resist. 2023 Feb 11;5(1):dlad012. doi: 10.1093/jacamr/dlad012 (PMC9921722; doi:10.1093/jacamr/dlad012)
Supplement: dlad012_Supplementary_Data [file dlad012_supplementary_data.docx]

**Supplementary Information: Sustained increases in antibiotic prescriptions per primary care consultation for upper respiratory tract infections in England during the COVID-19 pandemic**

This supplementary material is hosted by the Journal of Antimicrobial Chemotherapy – Antimicrobial Resistance as supporting information alongside the article *Sustained increases in antibiotic prescriptions per primary care consultation for upper respiratory tract infections in England during the COVID-19 pandemic*, on behalf of the authors, who remain responsible for the accuracy and appropriateness of the content. The same standards for ethics, copyright, attributions and permissions as for the article apply.

**Table S1**: Summary of characteristics of patients diagnosed with an upper respiratory tract infection in primary care in England, April 2014 – May 2022

|  | | **All patients (n=518,859)** | | **Patients prescribed an antibiotic (n=262,851)** | | **Patients not prescribed an antibiotic (n=256,008)** | |
| --- | --- | --- | --- | --- | --- | --- | --- |
| **Patient Characteristics** | | **Frequency** | **Proportion**  **(%)** | **Frequency** | **Proportion**  **(%)** | **Frequency** | **Proportion**  **(%)** |
| Sex | Female | 300,894 | 58.0 | 159,881 | 60.8 | 141,013 | 55.1 |
|  | Male | 216,793 | 41.8 | 102,377 | 38.9 | 114,416 | 44.7 |
|  | Unknown | 1,172 | 0.2 | 593 | 0.3 | 579 | 0.2 |
| Age-group (years) | 0-5 | 110,349 | 21.3 | 44,704 | 17 | 65,645 | 25.6 |
|  | 06-11 | 48,830 | 9.4 | 23,280 | 8.9 | 25,550 | 10.0 |
|  | 12-17 | 34,419 | 6.6 | 17,993 | 6.9 | 16,426 | 6.4 |
|  | 18-59 | 250,638 | 48.3 | 138,730 | 52.8 | 111,908 | 43.7 |
|  | 60-74 | 50,055 | 9.7 | 26,404 | 10.0 | 23,651 | 9.2 |
|  | 75+ | 24,568 | 4.7 | 11,740 | 4.5 | 12,828 | 5.0 |
| Region | London | 91,487 | 17.6 | 43,076 | 16.4 | 48,411 | 18.9 |
|  | Midlands and East of England | 171,150 | 33.0 | 89,521 | 34.1 | 81,629 | 31.9 |
|  | North of England | 118,556 | 22.8 | 64,954 | 24.7 | 53,602 | 20.9 |
|  | South of England | 137,666 | 26.5 | 65,300 | 24.8 | 72,366 | 28.3 |


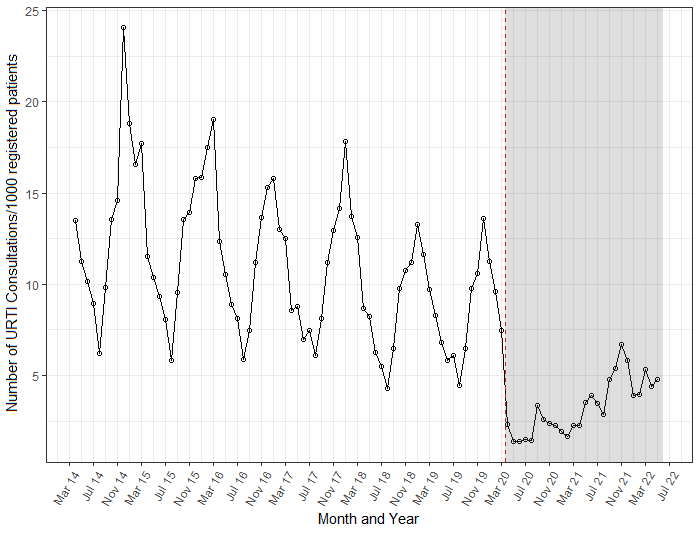


***Figure S1*:** Consultation rate for upper respiratory tract infections in primary care in England, per 1,000 registered patients, April 2014 to May 2022. (Grey area = COVID-19 pandemic period)

*
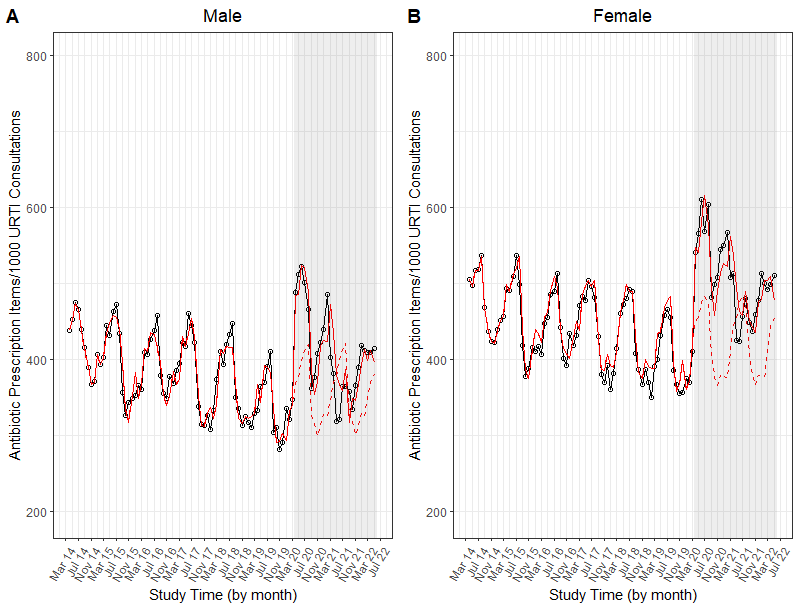
Figure S2.* Stratified Analysis by Sex: Antibiotic prescribing rate for URTI consultations in England, per 1000 URTI consultations (grey area = COVID-19 pandemic period; black line = observed data; solid red line = predicted values by ARIMA model; dashed red line = counterfactual predicted by ARIMA model in absence of COVID-19 pandemic)


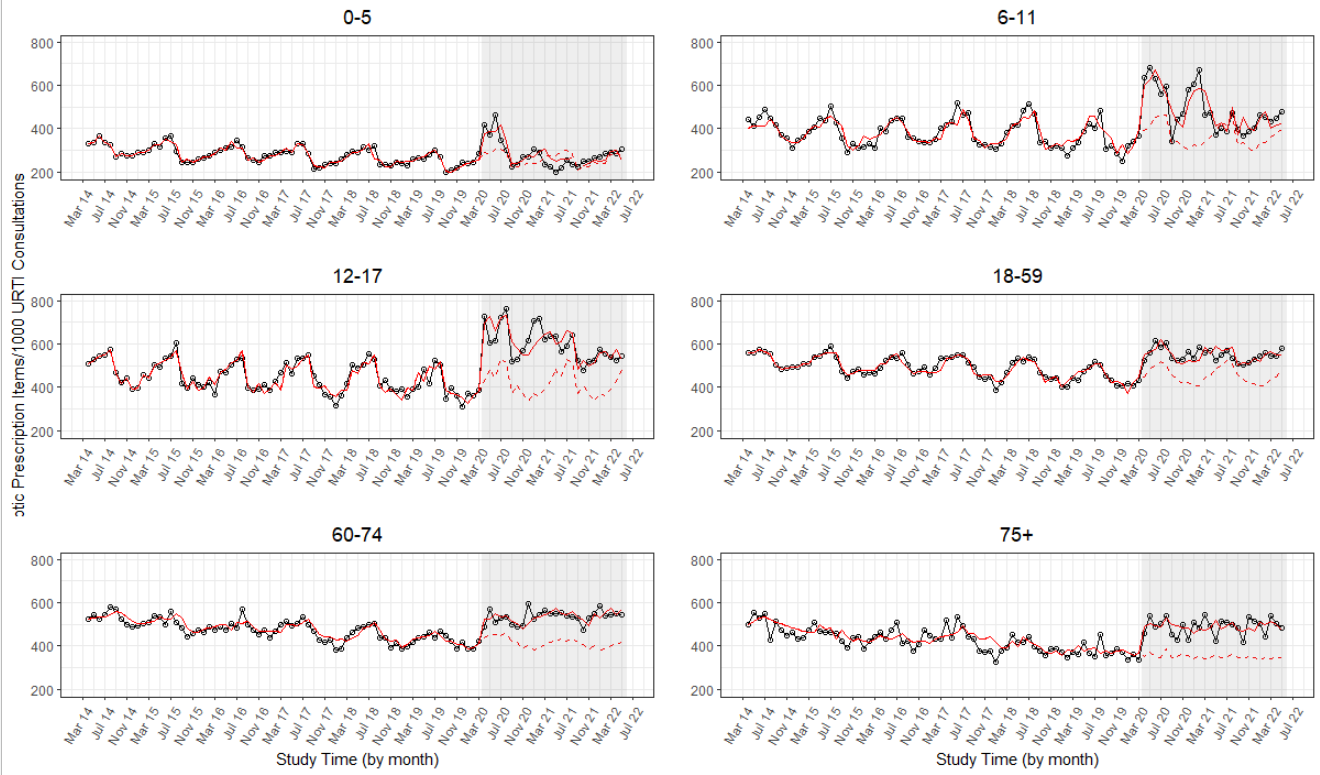
*Figure S3.* Stratified Analysis by Age Group: Antibiotic prescribing rate for URTI consultations in England, per 1000 URTI consultations (grey area = COVID-19 pandemic period; black line = observed data; solid red line = predicted values by ARIMA model; dashed red line = counterfactual predicted by ARIMA model in absence of COVID-19 pandemic)


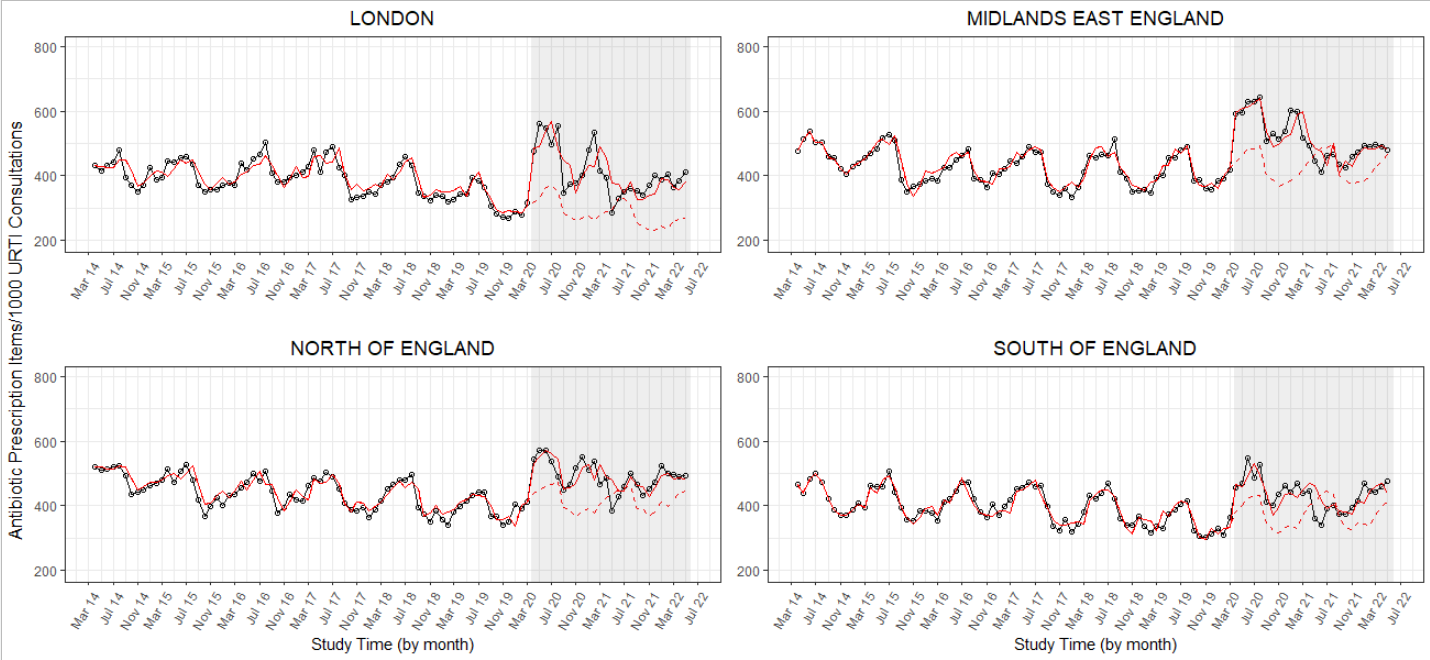
*Figure S4.* Stratified Analysis by Region: Antibiotic prescribing rate for URTI consultations in England, per 1000 URTI consultations (grey area = COVID-19 pandemic period; black line = observed data; solid red line = predicted values by ARIMA model; dashed red line = counterfactual predicted by ARIMA model in absence of COVID-19 pandemic)
